# Salmonella Tol-Pal Reduces Outer Membrane Glycerophospholipid Levels for Envelope Homeostasis and Survival during Bacteremia

**DOI:** 10.1128/IAI.00173-18

**Published:** 2018-06-21

**Authors:** Revathi Masilamani, Melina B. Cian, Zachary D. Dalebroux

**Affiliations:** a Department of Microbiology and Immunology, University of Oklahoma Health Sciences Center, Oklahoma City, Oklahoma, USA; University of California San Diego School of Medicine

**Keywords:** Intracellular, facultative, lysosome, vacuole, pathogen, macrophage, mouse, bacteria, systemic pathogenesis, Tol-Pal, outer membrane, anionic glycerophospholipids, phosphatidylglycerol, phosphatidylethanolamines, lipid, constriction, membrane curvature, trafficking, translocation, transport, virulence, pathogenesis, barrier, transenvelope complex, septation, ion channel, motor, proton-motive force, YbgC, TolQ, TolR, TolA, TolB, Pal, CpoB/YbgF, RcsF, salmonella, cell envelope, Gram-negative bacteria, peptidoglycan hydrolases, periplasm, phospholipids

## Abstract

Salmonellae regulate membrane lipids during infection, but the exact proteins and mechanisms that promote their survival during bacteremia remain largely unknown. Mutations in genes encoding the conserved Salmonella enterica serovar Typhimurium (*S*. Typhimurium) Tol-Pal apparatus caused the outer membrane (OM) sensor lipoprotein, RcsF, to become activated. The capsule activation phenotype for the mutants suggested that Tol-Pal might influence envelope lipid homeostasis. The mechanism involves reducing OM glycerophospholipid (GPL) levels, since the mutant salmonellae similarly accumulated phosphatidylglycerols (PGl) and phosphatidylethanolamines (PE) within the OM in comparison to the wild type. The data support the Escherichia coli model, whereby Tol-Pal directs retrograde GPL translocation across the periplasm. The *S*. Typhimurium mechanism involves contributions from YbgC, a cytoplasmic acyl coenzyme A (acyl-CoA) thioesterase, and CpoB, a periplasmic TolA-binding protein. The functional relationship between Tol-Pal and YbgC and CpoB was previously unresolved. The *S*. Typhimurium Tol-Pal proteins contribute similarly toward promoting OM-GPL homeostasis and Rcs signaling inactivity but differently toward promoting bacterial morphology, rifampin resistance, survival in macrophages, and survival in mice. For example, *tolQ*, *tolR*, *tolA*, and *cpoB* mutants were significantly more attenuated than *ybgC*, *tolB*, and *pal* mutants in a systemic mouse model of disease. Therefore, key roles exist for TolQ, TolR, TolA, and CpoB during murine bacteremia, which are independent of maintaining GPL homeostasis. The ability of TolQR to channel protons across the inner membrane (IM) is necessary for *S*. Typhimurium TolQRA function, since mutating conserved channel-facing residues rendered TolQ ineffective at rescuing deletion mutant phenotypes. Therefore, Tol-Pal promotes *S*. Typhimurium survival during bacteremia, in part, by reducing OM GPL concentrations, while TolQRA and CpoB enhance systemic virulence by additional mechanisms.

## INTRODUCTION

Nontyphoidal salmonellae, like Salmonella enterica serovar Typhimurium (*S*. Typhimurium), can inflict a severe bacteremia upon immune-deficient humans and inbred mice (1). During systemic pathogenesis, salmonellae predate immune phagocytes, such as macrophages (Mϕs), from within a phagolysosome vacuole (2–4). Vacuolar bacteria detect the acidic pH and cationic antimicrobial peptides (CAMPs) within the lumen and respond by remodeling the lipids within their outer membrane (OM) bilayer (5, 6).

**FIG 1:**
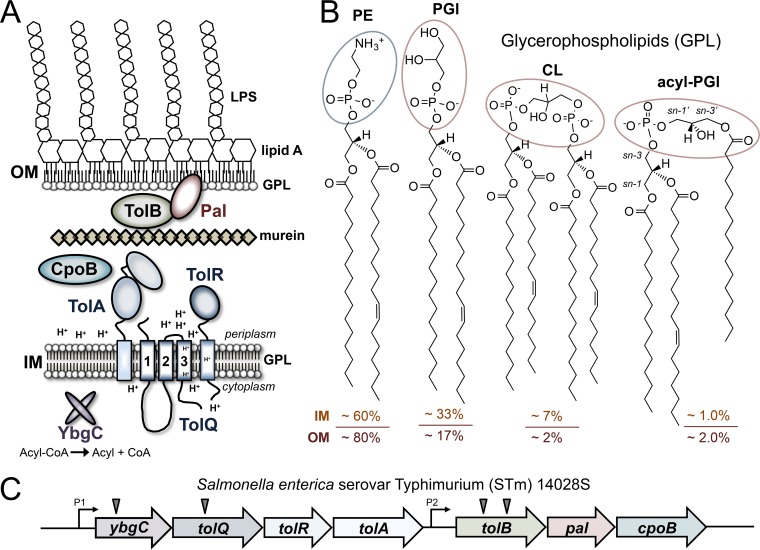
Tol-Pal spans the Gram-negative cell envelope, and salmonellae produce four major glycerophospholipid (GPL) families within their dual membranes. (A) Enteric bacteria produce the Tol-Pal system of proteins, which are present within the cytoplasm, periplasm, and each membrane of their envelope. The inner membrane (IM) consists exclusively of GPLs, while the outer membrane (OM) is asymmetric and consists mostly of inner-leaflet GPLs and outer-leaflet lipopolysaccharides (LPS). Gram-negative bacteria produce a murein sacculus or peptidoglycan exoskeleton, which is attached to the OM by lipoproteins. (B) Salmonella enterica serovar Typhimurium (*S*. Typhimurium) generates four major GPL families, which are distinguished by their polar head group structures, indicated by blue and red ovals. Four representative species are depicted for each family. Each species shares an *sn-1* esterified C_16:0_ and an *sn-2* esterified C_18:1_ acyl chain and is abundantly present within the *S*. Typhimurium envelope. The phosphatidylethanolamines (PEs) are diacylated, have a zwitterionic phosphoethanolamine head group, and constitute roughly 60% of the GPL within the IM and 80% of the GPL within the OM (9). Phosphatidylglycerols (PGls) are diacylated, have a singly anionic glycerol-phosphoglycerol head group, and comprise roughly 33% of the GPL within the IM and 17% of the GPL within the OM (9). Cardiolipins (CLs), or diphosphatidylglycerols (diPGls), are tetra-acylated, harbor doubly anionic glycerol-phosphoglycero-phosphoglycerol head group structures, and constitute roughly 7% of the GPL within the IM and 2% of the GPL within the OM (9). Acyl-PGls (aPGls) are triacylated, possess anionic *sn-3*′ esterified glycerol-phosphoglycerol head groups, and constitute roughly 1% of the GPL within the IM and 2% of the GPL within the OM (10, 65). (C) The enterobacterial Tol-Pal system is transcribed from at least two promoters as two operons, *ybgC-tolQRA-tolB-pal-cpoB* and *tolB-pal-cpoB* (28, 66). We previously identified transposon insertion mutations in *ybgC*, *tolQ*, and *tolB*, which caused RcsF-dependent *wza-lacZ* gene reporter activity to increase for *S*. Typhimurium (triangles) (47). The Rcs activation phenotype is manifested as a blue colony hue on indicator medium and is depicted in the present study for the site-directed *tol-pal* mutants (Fig. 4B).

The enterobacterial envelope consists of two concentric membranes, which are separated by an aqueous periplasmic space and a thin layer of peptidoglycan or murein (Fig. 1A) (7). The inner plasma membrane (IM) encases the cytoplasm and consists of glycerophospholipids (GPLs). The OM bilayer is asymmetric and consists mostly of GPLs within the inner leaflet and lipid A disaccharolipids within the outer leaflet (Fig. 1A). Lipid A amphiphiles anchor extended polar lipopolysaccharides (LPS) to the microbial surface through hydrophobic interactions with underlying GPL molecules (Fig. 1A) (5). Lipid asymmetry and LPS biochemistry provide intrinsic chemical barrier properties to the OM bilayer that protect Gram-negative bacteria against antibiotics and immune responses (8). Several OM lipid remodeling proteins and mechanisms are known for *S*. Typhimurium, but those that promote survival during systemic infection remain largely unknown (5).

Salmonellae produce four major GPL head group families and maintain a particular concentration and distribution for each within their dual bilayers (9). For example, zwitterionic phosphatidylethanolamines (PEs) predominate both membranes (Fig. 1B). Anionic phosphatidylglycerols (PGls), cardiolipins (CLs), and acyl-phosphatidylglycerols (aPGls) are increasingly less abundant (Fig. 1B) (9, 10). Interestingly, *S*. Typhimurium maintains roughly half as many PGls and CLs within its OM than within its IM (9, 10). Escherichia coli GPL anions concentrate at negatively curved regions of the plasma membrane and bind cytoskeletal proteins to influence division site placement (11, 12). Whether periplasmic GPL anions impact OM invagination and curvature during fission is not understood.

Proteobacterial lipoproteins are synthesized by the Lgt enzyme, which transfers diacylglyceryl (DAG) groups exclusively from PGl donor substrates to prolipoprotein acceptor substrates within the IM (13, 14). Select lipoproteins are then sorted, ferried across the periplasm, and inserted into the inner leaflet of the OM by the Lol system (15). The enterobacterial sensor lipoprotein, RcsF (regulator of capsule synthesis), and some other OM lipoproteins can adopt transmembrane (TM) configurations by luminal threading through β-barrel proteins (16) (17). Surface exposure permits electrostatic interactions between cationic RcsF residues and anionic LPS phosphates within the outer leaflet (18). Anionic charge disruption by CAMPs binding LPS molecules causes RcsF conformation to change. Then RcsF either fully relocalizes to the IM or extends across the periplasm to bind the multipass IM protein, IgaA (19) (16). IgaA is an essential repressor of two sensor kinases, RcsC and RcsD. When RcsF binds IgaA, RcsC and RcsD autophosphorylate and catalyze phosphotransfer to RcsA and RcsB (19) (20). The phosphorylated response regulators then bind to DNA and coordinate transcription of genes, including the *wza* operon, which encodes the proteins and enzymes for capsule synthesis and secretion (21). Synthetic lethal mutations and conditional depletion of E. coli PGls also cause Rcs signaling to increase, since RcsF accumulates within the IM (22, 23). Therefore, perturbing LPS structure and disrupting PGl homeostasis activate RcsF through slightly different mechanisms.

Proteobacteria commonly employ the Tol-Pal apparatus to promote OM barrier function, yet the biochemical mechanism is not fully understood (24). Enterobacterial Tol-Pal consists of seven proteins encoded by seven genes, which are part of two operons (Fig. 1A and C). The system contains a single cytoplasmic acyl coenzyme A (acyl-CoA) thioesterase enzyme, YbgC, which binds acyl carrier protein (ACP) (25–28). Remarkably, YbgC-ACP complexes bind PlsB and Pss, two key GPL biosynthesis enzymes (29). However, the role of YbgC in enterobacterial lipid homeostasis is not known. The remaining Tol-Pal proteins are better characterized. Each is noncatalytic, envelope associated, and localized to the division septum (30, 31). Septal Tol-Pal proteins promote OM constriction during fission by poorly understood biochemical mechanisms (30). The most well characterized mechanism involves TolQ and TolR, which form a proton channel across the plasma membrane to conduct proton motive force. Ion channel activity drives TolA, an IM-tethered periplasmic TolQR-binding protein, to change conformation, extend across the periplasm, and bind to Pal, an OM lipoprotein (Fig. 1A) (32–35). TolB is a periplasmic protein that normally binds and sequesters Pal from murein (Fig. 1A) (36, 37). Energized TolA displaces Pal from TolB and allows Pal to bind to septal murein, which causes the OM to invaginate (24, 30, 38, 39). Evidence suggests that TolQR activity might influence additional TolA mechanisms, which drive constriction at the septum (31, 40). For example, E. coli TolA binds CpoB/YbgF, a secreted periplasmic protein that is encoded by the last gene of the *tol-pal* operons (Fig. 1C) (28, 38, 39). *In vitro*, CpoB binds and inhibits lipoprotein (LpoB)-induced murein transpeptidase (Pbp1B) activity (31). However, the biological function of CpoB is unclear (28).

Salmonellae require *tolQ*, *tolR*, *tolA*, and *tolB* to maintain the OM barrier and to promote virulence (41–45). However, the biochemical function of Tol-Pal and the biological role of YbgC, Pal, and CpoB during murine bacteremia had not been determined. We provide data here to support that *S*. Typhimurium Tol-Pal functions biochemically, in part, to lower GPL levels within the OM. The *S*. Typhimurium data corroborate recent E. coli work and support the model whereby enterobacterial Tol-Pal mediates retrograde GPL translocation across the periplasm (46). Mutations in *S*. Typhimurium *tol-pal* genes cause equivalent increases in OM GPL levels but have variable phenotypic effects on *S*. Typhimurium morphology, antibiotic resistance, survival in phagocytes, and survival in mice. Therefore, Tol-Pal-driven OM GPL homeostasis only partially contributes to select phenotypes, including the promotion of *S*. Typhimurium survival in mice. The findings suggest that some Tol-Pal proteins, such as TolQRA and CpoB, execute additional biochemical mechanisms that are critical for *S*. Typhimurium survival during bacteremia.

## RESULTS

### Deleting *S*. Typhimurium *tol-pal* loci causes Rcs signaling activity to increase.

**FIG 2:**
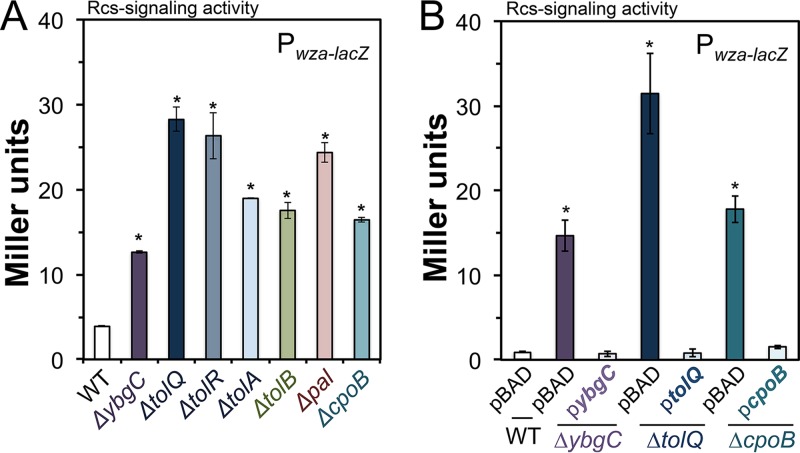
Deleting *S*. Typhimurium *tol-pal* genes causes Rcs signaling activity to increase. (A) The chromosomally integrated *wza-lacZ* gene reporter was used to quantify transcription, which resulted from Rcs signaling activity (47). Briefly, the bacteria were cultured in Luria-Bertani (LB) broth medium to an optical density at 600 nm (OD_600_) of between 0.6 and 0.8, or the mid-exponential growth phase (E phase). The cells were pelleted and lysed so that Miller units could be quantified by a standard β-galactosidase assay. Depicted are the average values ± standard deviation (SD) of results for five independent experiments. An asterisk indicates a statistically significant difference for the mutant relative to the wild type (WT). In all stated instances, two-tailed Student's *t* tests were used to determine significance (*P* < 0.05). (B) The *ybgC*, *cpoB*, and *tolQ* genes were cloned and expressed from pBAD24, alongside an empty vector (pBAD) control, in the wild-type and mutant *S*. Typhimurium genotypes. The bacteria carrying the plasmids were cultured in LB broth plus antibiotic until E phase. Basal pBAD promoter activity drove expression of the gene products. Rcs signaling activity was quantified by a β-galactosidase assay. The experiment was repeated three times, and the average values ± SD are shown. An asterisk indicates a statistically significant difference between the mutant and the wild type (*P* < 0.05).

We identified transposon insertions in *S*. Typhimurium *tol-pal* loci which caused RcsF signaling activity to increase (47). However, *S*. Typhimurium *tol-pal* mutants produce wild-type LPS molecules (45). Therefore, we reasoned that Tol-Pal might impact OM GPL levels. To test this hypothesis, we constructed seven individual deletion-insertion alleles for an *S*. Typhimurium 14028s genotype that carried a chromosomal copy of the *wza-lacZ* gene reporter (see Tables S1 and S2 in the supplemental material) (47). *S*. Typhimurium Tol-Pal promotes Rcs inactivity, since each mutant demonstrated significantly greater levels of *wza-lacZ* activity than the wild type (Fig. 2A). It was possible that the deletion-insertion alleles were causing polar effects on adjacent *tol-pal* genes (Fig. 1C), so we expressed *ybgC*, *tolQ*, and *cpoB*, in *trans*, for the respective mutant genotypes. Expressing the plasmid-borne alleles fully rescued the mutant phenotypes and decreased the *wza-lacZ* reporter levels to those of the wild type (Fig. 2B). Therefore, the deletion mutant phenotypes predominantly reflected the corresponding loss of the intended protein target.

### *S*. Typhimurium *ybgC*, *tolQ*, *tolR*, *tolA*, and *cpoB* mutants accumulate OM PGls and PEs.

**FIG 3:**
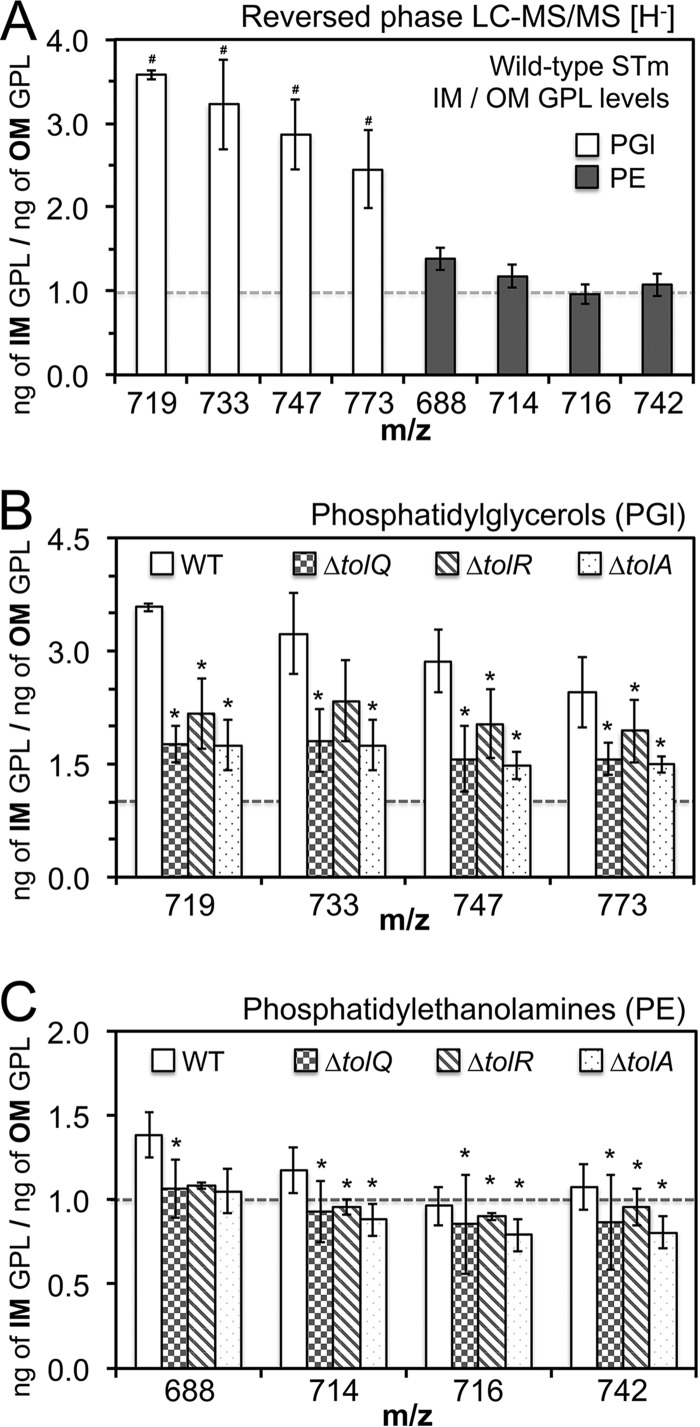
*tolQ*, *tolR*, and *tolA S*. Typhimurium mutants accumulate phosphatidylglycerols (PGls) and phosphatidylethanolamines (PEs) within the OM relative to the wild type. The IM and OM fractions for wild-type and mutant *S*. Typhimurium were isolated by sucrose density gradient ultracentrifugation (63). GPLs were extracted and quantified by reversed-phase liquid-chromatography tandem mass spectrometry (LC-MS/MS) (10). Purified PE and PGl molecules of defined molecular weight, structure, and concentration were used to generate standard curves. Four major *S*. Typhimurium PE and PGl molecules were then targeted for each membrane. The peak areas for the eight target molecules were compared to the standard curves to calculate the final ng/μl concentration in the membranes (Tables 1 and S3). (A) A graph depicts the IM/OM (ng/ng) ratio calculated for the wild type. The values were obtained using the ng/μl values of the four individual PE and PGl molecules. The average values ± SD were determined for three experiments. A number sign (#) indicates a statistically significant difference in the ng/μl concentration for IM versus the OM fraction of the wild-type *S*. Typhimurium envelope (*P* < 0.05) (Tables 1 and S3). The dashed line denotes a 1:1 ratio. (B) A bar graph depicts the IM/OM (ng/ng) ratio for the PGls within the wild-type and *tolQ*, *tolR*, and *tolA* mutant *S*. Typhimurium cell envelopes. An asterisk indicates a statistically significant difference in the OM PGl level (ng/μl) for the mutant relative to the wild type (*P* < 0.05) (Tables 1 and S3). (C) A graph depicts the IM/OM (ng/ng) ratio for the PEs within the wild-type and *tolQ*, *tolR*, and *tolA* mutant envelopes. An asterisk indicates a statistically significant difference in the OM PE level (ng/μl) for the mutant relative to the wild type (*P* < 0.05) (Tables 1 and S3).

The envelopes of the wild-type and the mutant *S*. Typhimurium were separated into a low-density IM fraction and a high-density OM fraction. The GPLs were extracted and quantified by liquid chromatography-tandem mass spectrometry (LC-MS/MS) (Fig. 3A; Tables 1 and S3) (10). Initially, we quantified the nanogram per microliter (ng/μl) concentrations of four phosphatidylglycerol (PGl) species and four phosphatidylethanolamine (PE) species for the wild-type *S*. Typhimurium. Consistent with decades-old biochemistry, *S*. Typhimurium maintains, statistically, between 2- and 4-fold fewer PGls within the OM than within the IM (Fig. 3A; Tables 1 and S3) (9). Minor variations were detected in the PE levels between the bilayers, but the differences were not significant (Tables 1 and S3). Recently, E. coli Tol-Pal was shown to mediate retrograde GPL translocation across the periplasm (46). Since TolQ, TolR, and TolA comprise the molecular workhorse for the enterobacterial Tol-Pal apparatus, we quantified the GPLs for the *tolQ*, *tolR*, and *tolA* mutants and compared them to those of the wild type (24). The IM GPL levels did not statistically vary between the mutants and the wild type (Table S3). However, the OM levels of three PGl species, *m/z* 719, 747, and 773, and three PE species, *m/z* 714, 716, and 742, were significantly increased for the *tolQ*, *tolR*, and *tolA* mutants (Fig. 3B and C; Table 1). Therefore, *S*. Typhimurium TolQRA promotes lower GPL levels within the OM.

**TABLE 1:** *tolQ*, *tolR*, and *tolA* mutant salmonellae accumulate PGl and PE molecules within the OM in comparison to the wild type^*a*^

*m/z* of GPL species	Level (ng/μl) (avg ± SD) of the individual GPL molecule in the OM fraction of:			
	WT	*tolQ* mutant	*tolR* mutant	*tolA* mutant
PGl				
719	22.43 ± 3.99^#^	34.53 ± 6.41*	33.72 ± 6.90*	35.26 ± 6.09*
733	1.53 ± 0.28^#^	1.71 ± 0.28*	2.14 ± 0.48	2.08 ± 0.44*
747	3.21 ± 0.70^#^	5.96 ± 1.38*	6.55 ± 2.03*	6.05 ± 1.35*
773	4.89 ± 0.86^#^	10.16 ± 1.39*	11.46 ± 2.92*	10.22 ± 1.27*
PE				
688	116.76 ± 15.35	133.01 ± 14.88*	129.50 ± 9.52	129.04 ± 14.61
714	35.43 ± 2.77	59.42 ± 3.85*	48.72 ± 2.84*	47.68 ± 1.67*
716	52.91 ± 6.79	67.15 ± 7.97*	69.50 ± 10.27*	66.60 ± 4.83*
742	39.23 ± 6.08	69.87 ± 7.16*	76.17 ± 11.12*	68.12 ± 4.32*

a Liquid chromatography-tandem mass spectrometry (LC-MS/MS) was used to quantify outer membrane glycerophospholipid (OM GPL) molecules. Overnight cultures of wild-type and *tolQ*, *tolR*, and *tolA* mutant salmonellae were back diluted 1:100 in 1 liter of LB broth and incubated at 37°C and 225 rpm for ∼3 h to mid-exponential growth phase. The cells were collected by centrifugation and resuspended in a sucrose solution, which began the osmotic spheroplasting and lysis procedure. The membranes were isolated by discontinuous sucrose density gradient ultracentrifugation (63). Three independent experiments were conducted, and the average values (ng/μl) ± standard deviation (SD) for these biological replicates are depicted. A number sign (#) indicates a statistically significant difference for wild-type *S*. Typhimurium in the level of the phosphatidylglycerol (PGl) molecule within the OM relative to the level of the PGl molecule within the IM, *P* < 0.05 (Fig. 3A; Table S3). An asterisk indicates a statistically significant difference in the level of the GPL molecule within the OM for the mutant relative to the level of the molecule within the OM of the wild type, *P* < 0.05 (Fig. 3B and C). *m/z*, mass-to-charge ratio; PE, phosphatidylethanolamine.

Unfortunately, E. coli *ybgC* and *cpoB* mutants were not tested for a GPL trafficking phenotype (46). Therefore, we measured OM GPL concentrations for *S*. Typhimurium *ybgC* and *cpoB* mutants relative to those of the wild type (Tables S4 and S5). As for the *tolQ*, *tolR*, and *tolA* mutants (Table 1), the OM concentrations of three PGl species, *m/z* 719, 747, and 773, and two PE species, *m/z* 714 and 742, were significantly greater for the *ybgC* mutants than for the wild type (Table S4). In fact, the fold increases in the OM GPL concentrations relative to those of the wild type were similar for each mutant that was tested, between 1.5- and 2.0-fold greater (Tables 1 and S4). The *cpoB* mutants consistently showed greater levels of PGls and PEs within the OM than did the wild type; however, only the levels of two PE species, *m/z* 714 and 742, were significantly greater (Table S5). Therefore, each *S*. Typhimurium Tol-Pal protein likely contributes similarly toward promoting fewer GPLs within the OM.

### Tol-Pal proteins variably promote *S*. Typhimurium morphology.

**FIG 4:**
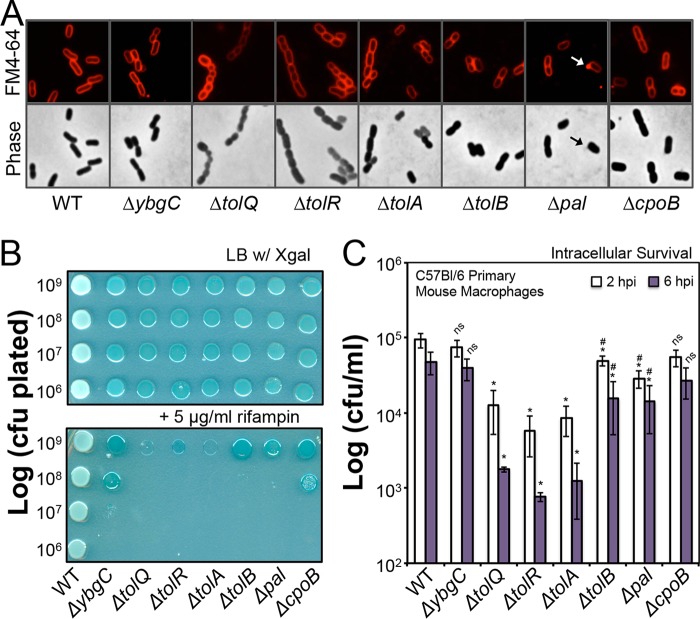
Salmonella Tol-Pal proteins differentially promote cell morphology, resistance to rifampin (Rif), and survival in primary mouse macrophages (Mϕs). (A) E-phase bacteria were fluorescently labeled with the lipopholilic membrane dye FM4-64 before being spotted onto agarose pads and visualized by phase-contrast and epifluorescence microscopy at ×100 magnification (47). An arrow indicates an FM4-64-stained vesicle emerging from *pal* mutant *S*. Typhimurium. Measurements were also made to determine whether the mutants were altered in their cell size relative to the wild type. For these measurements, we used the FM4-64 images and the fluorescently outlined bacteria (Table S6). Each mutant measured a reduced average cell length relative to the wild type. (B) Susceptibility to Rif was measured by normalizing E-phase bacteria to an OD_600_ of 1.0, or roughly 10^9^ CFU/ml, and spotting aliquots of serially diluted bacteria onto LB agar with or without Rif (5 μg/ml). Four independent experiments were executed, and one representative experiment is shown. The increase in Rcs signaling activity for the mutants is manifested as a blue colony color, which results from increased transcription of the *wza-lacZ* gene reporter. The increased LacZ levels in the bacteria result in increased cleavage of the indicator substrate, 5-bromo-4-chloro-3-indolyl-β-d-galactopyranoside, in the growth medium (X-Gal). (C) Primary C57BL/6 mouse bone marrow-derived macrophages (BMDMϕs) were infected with E-phase salmonellae at a multiplicity of infection (MOI) of roughly 10:1. Each strain was used to infect triplicate wells, and the surviving gentamicin-resistant intracellular CFU were enumerated at 2 and 6 hpi. Five independent experiments were performed. The average values ± SD for each time point are shown. An asterisk indicates a significant difference for the mutant relative to the wild type (*P* < 0.05). A number sign (#) indicates a significant difference for the *tolB* and *pal* mutants relative to the *tolQ* mutants (*P* < 0.05).

Given the seemingly cooperative role for each Tol-Pal component toward maintaining OM GPL homeostasis, we reasoned that the mechanism might influence other *S*. Typhimurium *tol-pal* phenotypes. E. coli *tolQRA* mutants grow as long chains of nonseptated bacilli in media of low osmolarity (30). In Luria-Bertani (LB) broth, wild-type *S*. Typhimurium grew as individual and pairs of rods (Fig. 4A). In contrast, *tolQ*, *tolR*, and *tolA* mutant *S*. Typhimurium grew as short chains (3 to 5 cells) of nonseptated rods and coccobacilli (Fig. 4A). The septation defect did not impact the doubling times, since like the wild type, each *tol-pal* mutant divided once every 23 to 25 min (Fig. S1). The *ybgC* mutants mostly grew as individual and pairs of rods, like the wild type (Fig. 4A). However, the *tolB*, *pal*, and *cpoB* mutant *S*. Typhimurium exhibited some chaining. Additionally, each of the seven mutant genotypes was shorter in length than the wild type (Table S6). Finally, as has been reported for *pal* mutant E. coli, *pal* mutant *S*. Typhimurium occasionally vesiculated (Fig. 4A) (30). Therefore, *S*. Typhimurium Tol-Pal proteins variably promote morphology and cell length, and TolQ, TolR, and TolA are the most critical.

### *S*. Typhimurium Tol-Pal proteins variably promote Rif resistance.

Rifampin (Rif) is a hydrophobic antibiotic that penetrates Gram-negative bacteria by diffusing through the OM (48). Lipid asymmetry and LPS biochemistry confer intrinsic Rif resistance to Enterobacteriaceae. Accordingly, Rif sensitivity typically reflects perturbations to OM lipid content and structure (48). To assess Tol-Pal's involvement in promoting Rif resistance, we assayed bacterial plating efficiency on LB agar with or without antibiotic (5 μg/ml) (Fig. 4B). The *tolQ*, *tolR*, and *tolA S*. Typhimurium mutants were the most sensitive to Rif, and zero CFU developed when as many as 10^9^ mutant salmonellae were spotted onto the medium with the antibiotic. The *tolB* and *pal* mutants were the next most sensitive, since plating nearly 10^8^ mutant cells resulted in zero CFU on LB agar plus Rif. Finally, *cpoB* and *ybgC* mutants were the least sensitive to Rif but were still more sensitive than the wild type. For instance, the wild type was not impacted when 10^6^ cells were spotted onto LB agar plus Rif (Fig. 4B). In contrast, plating 10^7^ *ybgC* and *cpoB* mutant cells resulted in severely impaired colony development for the *ybgC* mutant and no colony development for the *cpoB* mutant (Fig. 4B). Therefore, *S*. Typhimurium Tol-Pal proteins variably promote Rif resistance, and the TolQRA proteins are more critical than YbgC, TolB, Pal, and CpoB.

### Select Tol-Pal proteins are necessary for *S*. Typhimurium survival in primary Mϕs.

Salmonellae reside within the phagolysosome vacuoles of Mϕs during systemic pathogenesis in mice. Therefore, we infected primary bone marrow-derived Mϕs (BMDMϕs) from C57BL/6 mice with wild-type and *tol-pal* mutant bacteria. The capacity for intracellular survival was measured by enumerating gentamicin-resistant CFU/ml at 2 and 6 h postinfection (hpi). *S*. Typhimurium does not require YbgC or CpoB for survival in primary Mϕs, since *ybgC* and *cpoB* mutant levels were statistically identical to the wild-type levels at both time points (Fig. 4C). In contrast, the levels of *tolQ*, *tolR*, *tolA*, *tolB*, and *pal* mutant salmonellae were significantly less than the levels of the wild type for each time point. Furthermore, the *tolQ*, *tolR*, and *tolA* mutant levels were statistically less than the *tolB* and *pal* mutant levels (Fig. 4C). Therefore, *S*. Typhimurium TolQRA is more critical than TolB and Pal for bacterial survival in primary Mϕs, and YbgC and CpoB are not necessary under these conditions.

### Inducing TolQ translation, in *cis*, restores Rcs signaling inactivity, cellular morphology, Rif resistance, and intracellular survival to *tolQ* mutant *S*. Typhimurium.

**FIG 5:**
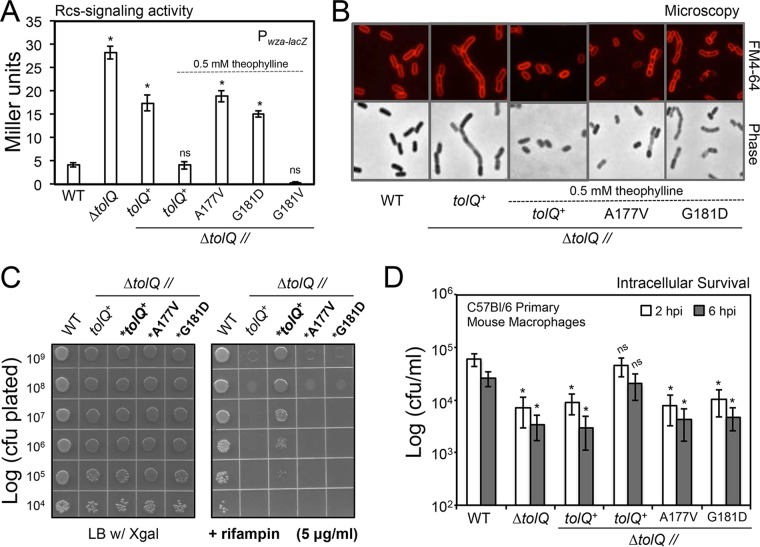
TolQ ion channel residues are necessary for function. (A) The rescue genotypes harbor a second-site chromosomal copy of the wild type or the mutant *tolQ* alleles (Fig. S2A and Table S1). Theophylline was added as the small-molecule inducer, which relieves translational repression of the riboswitch upstream of the second-site *tolQ* allele. The inducer was added at a 0.5 mM final concentration to the LB broth medium where indicated. The levels of Rcs signaling were quantified as Miller units from the gene reporter using E-phase cultures. The average Miller unit values ± SD for five independent experiments are depicted. ns, not significant. (B) Phase-contrast and epifluorescence microscopy was conducted using E-phase cells cultured with or without theophylline. (C) Rifampin susceptibility was determined using *S*. Typhimurium cultured to the E phase with or without theophylline. An asterisk indicates a bacterial genotype that was cultured with theophylline to the E phase in LB broth media. The inducer was also added to the agar medium in all instances. Four independent experiments were executed, and one representative experiment is shown. (D) Intracellular survival was measured using bacteria cultured to the E phase with or without theophylline. The first three genotypes from left to right on the *x* axis were cultured to the E phase in LB broth media without inducer. The inducer was added during culture in broth, as well as to the tissue culture medium during the infection period, for the final three genotypes depicted on the *x* axis. Five independent experiments were performed, and the average values ± SD for each time point are shown. An asterisk indicates a statistically significant difference for the mutant genotype relative to the wild type (*P* < 0.05).

The TolQ multipass transmembrane protein is the major subunit for the TolQR proton channel (32, 34, 35). To test the contribution of the TolQ polypeptide to the Δ*tolQ* mutant phenotypes, we cloned an independent copy of the wild-type *tolQ^+^* allele behind a constitutive promoter and a translational repressor, known as a riboswitch. This allele was then inserted at a neutral locus on the Δ*tolQ* mutant genome (Fig. S2 and Table S1) (49, 50). The riboswitch partially repressed translation of TolQ at the second site, since without the small-molecule inducer, theophylline, the Δ*tolQ//tolQ*^*+*^ bacteria were phenotypically similar to the Δ*tolQ* bacteria, which lacked the second chromosomal copy. This was true for Rcs signaling activity, cell morphology, Rif resistance, and intracellular survival (Fig. 5A to D). In contrast, adding theophylline rescued the deletion mutant phenotypes by reducing Rcs activity, restoring cell morphology, increasing Rif resistance, and enhancing intracellular survival of the Δ*tolQ//tolQ*^*+*^ genotype (Fig. 5A to D). Thus, *tolQ* mutant phenotypes are caused primarily by the loss of TolQ expression.

### Conserved TolQ ion channel residues are necessary for TolQRA function.

Residues within TM3 for E. coli TolQ interact with residues within the single TM of TolR (32, 51). Channel-facing residues facilitate movement of protons across the IM (Fig. 1A) (35). Mutating the E. coli TolQ alanine at position 177 to valine and the glycine at position 181 to glutamate severely limits TolQR's ability to energize TolA and thus impairs TolA's ability to bind Pal (35). Several residues are conserved for *S*. Typhimurium TolQ, including A177 and G181 (Fig. S2). Therefore, substitution mutant alleles, *tolQ^A177V^* and *tolQ^G181D^*, were engineered and inserted downstream of the riboswitch on the mutant genome (Fig. S2). Compared to the wild-type and the theophylline-induced Δ*tolQ//tolQ*^*+*^ rescued bacteria, the theophylline-induced Δ*tolQ//tolQ*^*A177V*^ and Δ*tolQ//tolQ*^*G181D*^ bacteria were highly attenuated and phenotypically identical to the uninduced negative-control bacteria, Δ*tolQ//tolQ*^*+*^. As a control for specificity, we engineered a neutral substitution allele, *tolQ^G181V^*, on the mutant genome. Inducing expression of the neutral TolQ substitution mutant protein fully reduced the Rcs signaling activity for the Δ*tolQ//tolQ*^*G181V*^ bacteria. This indicated that the negative phenotypic effect of expressing the mutant TolQ protein was specific to the G181D substitution, which likely perturbs TolQ function by imparting an electronegative charge to the channel lumen (Fig. 5A) (35, 51). Therefore, *S*. Typhimurium TolQ ion channel activity is necessary for TolQRA function.

### Salmonellae require each Tol-Pal component to survive during bacteremia, but TolQRA and CpoB are the most critical.

**FIG 6:**
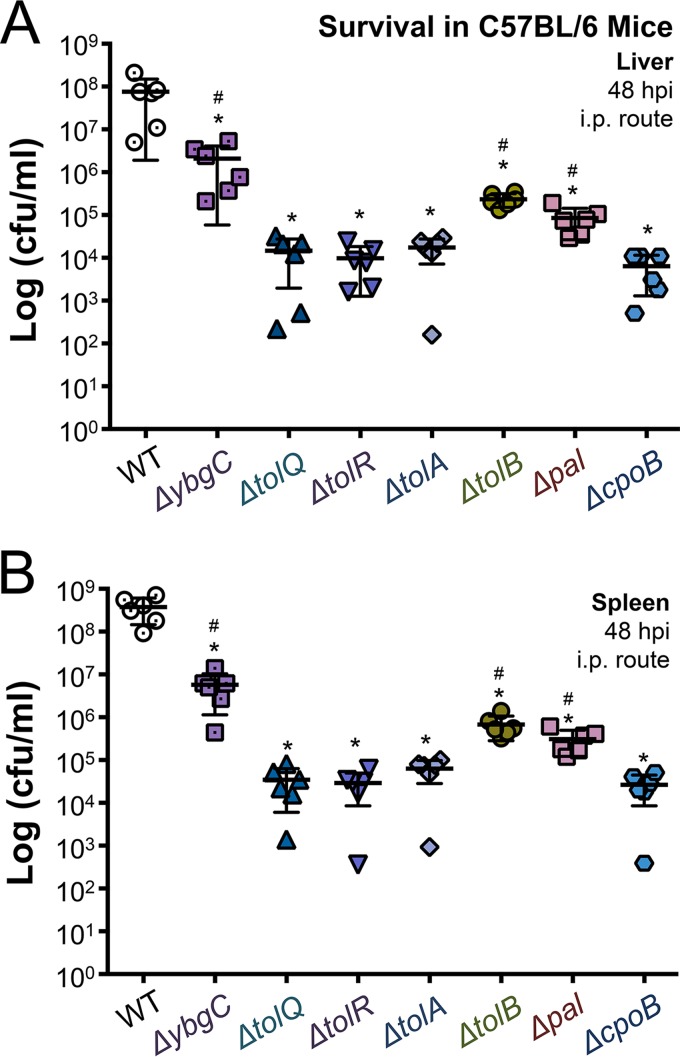
Tol-Pal proteins differentially promote *S*. Typhimurium survival during bacteremia. Groups of six female C57BL/6 mice were intraperitoneally (i.p.) injected with roughly 2.5 × 10^5^ wild-type or mutant salmonellae. The mice were euthanized at 48 h. The spleens and livers were dissected and homogenized. Samples were serially diluted, and bacterial CFU/ml were enumerated on LB agar with the appropriate antibiotic. (A) The CFU/ml levels were determined from the livers of the six individual mice infected with each strain. (B) The CFU/ml levels were determined from the spleens of the six individual mice infected with each genotype. An asterisk indicates a statistically significant difference relative to the wild type (*P* < 0.05). A number sign (#) indicates a significant difference relative to the *tolQ* mutant genotype (*P* < 0.05).

Salmonellae encounter a variety of host-killing mechanisms during systemic pathogenesis. Therefore, we reasoned that *S*. Typhimurium Tol-Pal proteins might differentially promote survival during murine bacteremia. Six female C57BL/6 mice were intraperitoneally inoculated with 10^5^ CFU of either wild-type or *tol-pal* mutant *S*. Typhimurium. The livers and spleens were dissected and homogenized at 48 hpi, and the numbers of CFU/ml of tissue were counted. During the infection, the wild type proliferated to levels between 10^7^ and 10^9^ CFU/ml at harvest (Fig. 6A and B). In contrast, statistically fewer surviving salmonellae were enumerated from the mice infected with each of the seven *tol-pal* mutant genotypes of *S*. Typhimurium than those infected with the wild type. Specifically, *tolQ*, *tolR*, and *tolA* mutants measured between 10^4^ and 10^5^ CFU/ml at harvest (Fig. 6B). The *tolB* and *pal* mutants were slightly less attenuated and measured between 10^5^ and 10^6^ CFU/ml (Fig. 6A and B). Though these mutants were not attenuated in phagocytes, statistically fewer *ybgC* and *cpoB* mutant salmonellae were recovered from the mice than the wild type. The *ybgC* mutant bacteria were the least attenuated, and between 10^6^ and 10^7^ CFU/ml were recovered (Fig. 6A and B). The *cpoB* mutants, in contrast, were as attenuated as the *tolQ*, *tolR*, and *tolA* mutants and measured between 10^4^ and 10^5^ CFU/ml at harvest (Fig. 6A and B). Therefore, each Tol-Pal protein contributes to *S*. Typhimurium survival during bacteremia, but TolQRA and CpoB are more critical than YbgC, TolB, and Pal under these conditions.

## DISCUSSION

This work establishes that the enterobacterial Tol-Pal system functions to reduce OM GPL levels, possibly by directing retrograde GPL translocation across the periplasm (46). *S*. Typhimurium typically maintains fewer PGls within the OM than within the IM (Fig. 3A and Table 1; see Table S3 in the supplemental material). Therefore, accumulating OM PGls likely has physiologic consequences (Fig. 3B and Table 1). In fact, the loss of each *S*. Typhimurium Tol-Pal protein impacted Rcs signaling activity, cell morphology, Rif resistance, and survival during bacteremia, including loss of YbgC and CpoB, whose biological relationship with Tol-Pal was unresolved (Fig. 2A, 4A and B, and 6A and B). *S*. Typhimurium Tol-Pal proteins likely participate in biochemical mechanisms in addition to GPL trafficking to enhance bacterial virulence, since *tolQ*, *tolR*, *tolA*, and *cpoB S*. Typhimurium mutants were more highly attenuated than *ybgC*, *tolB*, and *pal* mutants during bacteremia, yet *ybgC*, *tolQ*, *tolR*, *tolA*, and *cpoB* mutants measure similar increases in OM GPL concentrations (Table 1; Tables S3 to S5). Therefore, Tol-Pal-mediated reductions in OM GPLs only partially contributes to the *tol-pal* mutant phenotypes.

### Does Tol-Pal directly or indirectly influence OM-GPL trafficking?

The proteins and mechanisms required for Gram-negative bacteria to transport GPLs across the periplasm have eluded scientific discovery until recently (47, 52, 53). We now understand that some mechanisms involve multicomponent protein systems that span the envelope and derive energy from the IM in order to move GPLs across the periplasm. For example, the conserved Mla system (maintenance of lipid asymmetry) relies upon cytosolic ATP hydrolysis and periplasmic GPL-binding proteins to ferry GPLs across the periplasm (52). Whether Tol-Pal proteins directly bind GPLs is not known. In support of a direct binding mechanism, TolA promiscuously binds multiple OM lipoproteins, periplasmic proteins, colicins, and even phage particles (32). Conceivably, TolA binds GPL, or another GPL-binding protein, to direct GPL movement across the periplasm. The mechanism could also be indirect. For example, Tol-Pal membrane contact sites could facilitate GPL movement by influencing the activity of other GPL trafficking proteins and their mechanisms (36, 37). Analogously, Tol-Pal might promote formation of membrane hemifusions between the IM outer leaflet and OM inner leaflet. These intermediate bilayers could be conduits for GPL trafficking between the periplasmic leaflets of two segregated bilayers (Fig. 1A) (54). Future biochemical work is necessary to deduce the exact biochemical mechanism.

### What is the biological role of *S*. Typhimurium limiting OM PGl concentrations?

Salmonellae maintain two to four times fewer PGls within the OM than within the IM, but the mechanism and significance are not known (Fig. 3A; Tables 1 and S3) (9). The levels of particular PGl and PE molecules were similarly increased for the *ybgC*, *tolQ*, *tolR*, and *tolA* mutants in comparison to those of the wild type (Fig. 3B and C; Tables 1 and S4). However, the *ybgC* mutant Rif sensitivity and virulence attenuation were far less severe than those for the *tolQ*, *tolR*, and *tolA* mutants (Fig. 4B and 6A and B). Thus, reducing OM GPLs only partially impacts *S*. Typhimurium *tol-pal* phenotypes, and additional biochemical mechanisms of Tol-Pal are necessary to fully promote antimicrobial resistance and virulence.

Regarding the biological role for *S*. Typhimurium limiting OM PGls, OM PGls might be binding receptors for toxic ligands. Alternatively, accumulating OM PGls might render resistance determinants, such as LPS and porins, ineffective. In support of PGls acting as receptors for antibiotics, spontaneous Gram-positive resistance to daptomycin, a cyclic lipopeptide antibiotic, involves mutations that cause three biochemical phenotypes related to membrane PGl homeostasis. These include loss of PGl synthesis, neutralization of anionic PGl head groups by the addition of lysine, and inversion of lysinylated PGl molecules into the outer leaflet of the plasma membrane (55, 56). By analogy, *S*. Typhimurium might maintain resistance to select agents by limiting the concentration and/or altering the chemical structure of PGl head groups within the OM. In support of this hypothesis, we previously demonstrated that PagP, a conserved enterobacterial OM phospholipase A1/palmitoyltransferase enzyme, limits *S*. Typhimurium OM PGl levels by transferring palmitoyl groups from inverted GPL donor substrates to inverted PGl acceptor substrates within the OM outer leaflet (10). Palmitoylating PGl head groups might prevent cathelicidin antimicrobial peptides from inserting into the *S*. Typhimurium bilayer, since PagP enhances salmonella resistance to these toxins (57). Finally, PGls are the principal donor substrates for lipoprotein biosynthesis (14). Therefore, Tol-Pal-mediated PGl trafficking might influence OM lipoprotein transport in a manner that impacts *S*. Typhimurium morphology, Rif resistance, and survival during infection.

### What is the biochemical role of YbgC and CpoB during systemic infection?

E. coli YbgC interacts with multiple cytoplasmic proteins, which have biochemical roles in GPL metabolism. The interacting proteins include acyl carrier protein (ACP), *sn*-glycerol-3-phosphate acyltransferase (PlsB), and phosphatidylserine synthase (Pss) (29). The functional consequence of the YbgC interactions with these proteins is not known. The Pss enzyme is essential for enterobacterial viability and necessary for PE synthesis (58). *S*. Typhimurium *ybgC* mutants accumulate OM PGl and PE molecules (Table S4). Therefore, it will be interesting to determine whether YbgC thioesterase activity, or the YbgC-protein interactions, contribute to OM GPL homeostasis and virulence for *S*. Typhimurium.

The exact biochemical role of CpoB during *S*. Typhimurium infection is more difficult to predict given the presumed absence of catalytic activity. For E. coli, CpoB interacts with TolA, but *cpoB* mutants do not exhibit Tol-Pal phenotypes (28, 59). Recently, CpoB was implicated in altering peptidoglycan cross-linking for septation (31). This was determined using *in vitro* protein interaction analysis and LpoB-induced Pbp1B-murein transpeptidation reactions. However, no clear septation or murein cross-linking defect has been demonstrated for E. coli *cpoB* mutants (28, 31). Therefore, the function of CpoB and the significance of the TolA interaction have not been fully elucidated. We show that CpoB promotes reduced *S*. Typhimurium Rcs signaling, reduced levels of particular OM GPLs, increased cell length, increased Rif resistance, and increased survival in mice (Fig. 2A and B, 4A and B, and 6A and B; Tables S5 and S6). In fact, the TolQ, TolR, TolA, and CpoB proteins were more critical in mice than the YbgC, TolB, and Pal proteins (Fig. 6A and B). In contrast, loss of CpoB did not influence *S*. Typhimurium survival in primary Mϕs, but TolQ, TolR, and TolA were critical under these conditions (Fig. 4C). We postulate that during systemic pathogenesis, CpoB executes a key biochemical mechanism. It is possible that the mechanism involves TolQRA. Alternatively, CpoB functions independently of Tol-Pal to promote survival in mice. The binding interfaces for the TolA-CpoB interaction have been reported (59). Therefore, it should be possible to determine the functional role of the predicted *S*. Typhimurium CpoB-TolA interaction toward the pathogenesis phenotype.

Many types of Gram-negative bacteria harbor the Tol-Pal system. In the case of *S*. Typhimurium, one common biological function of the seven Tol-Pal proteins is to promote survival during systemic pathogenesis in mice. Whether or not CpoB or YbgC works as part of the Tol-Pal apparatus during bacteremia remains an open question. Further, the exact host-killing mechanisms that restrict *tol-pal* mutants in Mϕs and mice are not known. These data will provide key insight into the biochemical involvement of *S*. Typhimurium Tol-Pal during infection.

## MATERIALS AND METHODS

For the materials used in this study, see Materials and Methods in the supplemental material.

### Bacterial strains, growth conditions, and genetics.

The wild-type Salmonella enterica serovar Typhimurium (*S*. Typhimurium) bacteria were derived from the 14028s genotype and contain a chromosomally integrated *wza-lacZ* gene promoter fusion (Table S1) (60). Salmonellae were typically grown in Luria-Bertani (LB) broth medium on a roller drum with aeration at 37°C overnight (OVN) or to the mid-exponential growth phase (E phase) (optical density at 600 nm [OD_600_], 0.6 to 0.8). E-phase bacteria were obtained by a 1:100 back dilution of an OVN culture, followed by a 3- to 4-h incubation period. Plasmid-bearing bacteria were grown with the respective antibiotics as follows: ampicillin, 100 μg/ml; kanamycin, 50 μg/ml; tetracycline, 10 μg/ml; and chloramphenicol, 25 μg/ml. All the deletion-insertion mutants were created using the phage lambda red recombinase system (61). The deletion-insertion alleles were horizontally transferred to fresh non-pKD46-bearing wild-type *wza-lacZ* salmonellae using bacteriophage P22 HT105/1 *int*-201. Complementation of *tolQ* at *att*Tn*7*, which is 3′ of *glmS* on the *S*. Typhimurium genome, was achieved using the pGRG37 vector (49). The transgene was introduced into the pBAV1k vector, and *tolQ* alleles were positioned 3′ of a T5 promoter and a riboswitch by Gibson Assembly (50, 62). Subsequently, the T5-riboswitch-*tolQ* expression allele was amplified and cloned into pGRG37 using the PacI and XhoI restriction enzyme sites. Second-site TolQ complementation was achieved by adding 0.5 mM theophylline. Site-directed amino acid substitutions were created using the pBAV1K-*tolQ* plasmid as a template. PCR mutagenesis was executed using purified primers from Integrated DNA Technologies (IDT) (Table S2). Point mutations were placed at the *att*Tn7 integration site as described above.

### β-Galactosidase assay and microscopy.

E-phase bacteria were pelleted and resuspended in Z-buffer, and OD_600_ readings were taken to quantify culture density. Bacteria were permeabilized with chloroform and 0.1% (wt/vol) SDS. The LacZ substrate ONPG (ο-nitrophenyl-β-d-galactoside) was added (4 mg/ml), and the whole-cell lysates were incubated at 28°C until a yellow color was observed. Na_2_CO_3_ at 1 M was added as a stop solution, and the mixture was centrifuged. The supernatant was read at OD_420_, and β-galactosidase activity (Miller units) was calculated using the following equation: (1,000 · OD_420_)/(time · volume · OD_600_). For live-cell imaging, E-phase phase bacteria were labeled with FM4-64 (0.5 μg/ml), a lipophilic membrane dye, in parallel for 1 h at 37°C in LB broth. Agarose pads were made using a 0.7% agarose–0.5 μg/ml FM4-64 phosphate-buffered saline (PBS) solution and gently placed on the slides. FM4-64-labeled cells were spotted onto the pads and air dried. A coverslip was placed on the slide, and the sides were sealed with a hot-glue gun. The slides were visualized under ×100 magnification by phase-contrast and epifluorescence microscopy.

### Rif sensitivity assay.

Plating efficiency on rifampin (Rif) was determined by first adjusting the density of E-phase bacterial cultures to an OD_600_ of 1.0 or roughly 1.0 × 10^9^ CFU/ml. The cells were then serially diluted 10-fold in PBS. Aliquots were spotted onto LB agar alone or LB agar plus Rif (5 μg/ml). Two microliters of each bacterial suspension was spotted onto the plates, and the plates were dried and incubated at 37°C OVN. For the *tolQ* mutant rescue experiments, 0.5 mM theophylline was added to the LB broth and the LB agar medium where stated in the figure legend (Fig. 5C).

### Infection of primary mouse Mϕs.

Primary bone marrow-derived mouse Mϕs (BMDMϕs) were prepared by standard methods after harvesting the marrow from the femurs of 6- to 8-week-old female C57BL/6 mice that were purchased from Jackson Laboratories. Macrophage colony-stimulating factor (M-CSF) was prepared from the supernatants of stably transfected NIH 3T3 immortalized fibroblast cells. The Mϕs were differentiated in BMDMϕ medium (RPMI 1640 with 20% fetal bovine serum [FBS], 10% M-CSF, 1% l-glutamine, 1% sodium pyruvate, 1% penicillin-streptomycin, and 50 μM β-mercaptoethanol). Differentiated Mϕs were removed after 5 to 7 days of incubation at 37°C with 5% CO_2_. The Mϕs were then plated at 2.5 × 10^5^ cells/ml in each well of a 24-well plate and allowed to adhere OVN. E-phase bacteria were diluted in Mϕ medium (RPMI 1640, 20% FBS, 1% l-glutamine, and 50 μM β-mercaptoethanol) and added to the monolayers at a 10:1 multiplicity of infection. Triplicate wells were infected for each genotype, and surviving intracellular CFU were enumerated at 2 and 6 hpi. (For additional details, see Materials and Methods in the supplemental material.)

### Membrane fractionation, protein quantification, and GPL extraction.

OVN cultures of wild-type and *tolQ*, *tolR*, and *tolA* mutant *S*. Typhimurium bacteria were back diluted 1:100 in 1 liter of LB broth and incubated at 37°C and 225 rpm for ∼3 h. The cells were collected by centrifugation and resuspended in a sucrose solution to begin the osmotic spheroplasting and lysis procedure, which allows for the efficient separation of the bilayers. The IM and OM were isolated using a discontinuous sucrose density gradient and ultracentrifugation (63). The membranes were resuspended in 1 ml of 1 mM Tris, pH 7.5, and stored at −20°C. Membrane protein concentrations were measured using Pierce Coomassie Plus Bradford assay reagent (Thermo Scientific). Equivalent (500 μg of protein) amounts of membrane were extracted to collect glycerophospholipids (GPLs) by the Bligh-Dyer method (64). The purified GPLs were dried under N_2_ and resuspended in 300 μl of solution of 2:3:1 CHCl_3_-MeOH-H_2_O (where MeOH is methanol). (For additional details, see Materials and Methods in the supplemental material.)

## Supplementary Material

Supplemental material
