# Supplementary material for: Salmonella Tol-Pal Reduces Outer Membrane Glycerophospholipid Levels for Envelope Homeostasis and Survival during Bacteremia
Source: Infect Immun. 2018 Jun 21;86(7):e00173-18. doi: 10.1128/IAI.00173-18 (PMC6013679; doi:10.1128/IAI.00173-18)
Supplement: Supplemental material [file supp_86_7_e00173-18__index.html]

Supplemental material 

# Salmonella Tol-Pal Reduces Outer Membrane Glycerophospholipid Levels for Envelope Homeostasis and Survival during Bacteremia

## Supplemental material

- Supplemental file 1 -

  Supplemental materials and methods. Table S1. Bacterial strains used in this study. Table S2. Primers used in this study. Table S3. *tolQ*, *tolR*, and *tolA* mutant *S.* Typhimurium does not measure significant differences in IM GPL levels. Table S4. *S.* Typhimurium *ybgC* mutants accumulate PGLs and PEs within the OM relative to the wild type. Table S5. *S.* Typhimurium *cpoB* mutants generally accumulate PGLs and PEs within the OM relative to the wild type, but only minor significant differences are measurable. Table S6. *S.* Typhimurium *tol-pal* mutants are shorter than the wild type. Fig. S1. *tol-pal* mutants divide at rates comparable to wild-type *S.* Typhimurium in Luria-Bertani broth media. Fig. S2. Schematic depicting the *tolQ* mutant complementation strategy, which we used to test the functional role of conserved TolQ channel residues within the third transmembrane helix of the protein.

  PDF, 969K
