# Supplementary material for: Salmonella Tol-Pal Reduces Outer Membrane Glycerophospholipid Levels for Envelope Homeostasis and Survival during Bacteremia
Source: Infect Immun. 2018 Jun 21;86(7):e00173-18. doi: 10.1128/IAI.00173-18 (PMC6013679; doi:10.1128/IAI.00173-18)
Supplement: Supplemental material [file IAI.00173-18_zii999092462s1.pdf]

SUPPORTING MATERIALS AND METHODS

Materials. Restriction enzymes were obtained from New England Biolabs and T4 DNA ligase was obtained from Promega (Madison, USA). PCR reactions were carried out using Accuprime pfx polymerase (Invitrogen). X-gal (5-bromo-4-chloro-3-indolyl-β-D-galactopyranoside) was obtained from Biotium, USA. ONPG (*o*-nitrophenyl-β-D-galactoside) was purchased from Sigma-Aldrich. Luria-Bertani (LB) medium and arabinose were purchased from BD Biosciences. Sucrose and glucose were purchased from British Drug Houses analytical (BDH) chemicals. Theophylline was procured from Tokyo Chemical Industry (TCI) America, USA. Fetal bovine serum (FBS) was purchased from Atlanta Biologicals, USA and all the cell culture media and antibiotics were obtained from Corning Inc.

Infection of primary mouse macrophages (continued from the main text). The plates were centrifuged at 500 rpm for 10 min to allow the bacterial cells to contact the monolayers, and the bacteria were incubated with the Mϕs for 45 min at 37°C. Next, the wells were washed with wash medium (RPMI 1640), then the Mϕ media was added with 100 μg/ml gentamycin to kill extracellular bacteria, and the infected cells were incubated until two hours. For the wells representing the six-hour time point, the media was removed and replaced with Mϕ media at 10 μg/ml gentamycin. For harvesting, wells were washed with 1 ml wash medium three times and Mϕs were lysed using 0.1% tritonX-100 and surviving intracellular cfu were enumerated on LB agar.

Membrane fractionation, protein quantification, and glycerophospholipid (GPL) extraction (continued from the main text). The GPL were mixed 1:1 with MeOH and 30 µl was loaded into glass inserts within vials and introduced to the Sample Manager for the Waters Acquity UPLC console, which was interfaced with a Phenomenex Luna 5 µ phenyl-hexyl column (75 Å~ 4.66 mm). The flow through needle delivered 5 µl injections for each membrane extract over the column. Dual injection sequences were run to obtain two measurements for each extract, which were ultimately averaged. Curves were generated using commercial standards as described previously (1). To control for differences in GPL extraction, the same work up and extraction procedure was conducted twice on two independent days for each membrane and the resulting values for each biological replicate represent two independent extractions, as well as the average of two injections. Furthermore, three independent biological replicates were examined. Glycerophospholipid retention was achieved at a flow rate of 0.35 mL/min using mobile phase A, consisting of CH_3OH, and mobile phase B consisting of water. A three-step gradient used started at 100% A for 1 min, continued at 100-50% A over the next 3 min, was held at 50% A for 4 min, returned to starting conditions in 0.1 min, and was allowed to equilibrate for an additional 3 min for a total run time of ~ 11.10 min. The samples were delivered to the Applied Biosystems Sciex API 4000 LC/MS/MS Triple Quad Mass Spectrometer. The instrumental settings used to target and quantify the individual GPL molecules were the same as described (1).

SUPPORTING TABLES

Table S1. Bacterial strains used in this study

**Strain or plasmid**	**Genotype and Notes**	**Reference**
Salmonella enterica serovar Typhimurium 14028s		
ZD004	Wild-type, *wza::lacZ::cat*	(2)
ZD005	*ΔybgC::tetRA, wza::lacZ::cat*	This study
ZD006	*ΔtolQ::tetRA, wza::lacZ::cat*	This study
ZD007	*ΔtolR::tetRA, wza::lacZ::cat*	This study
ZD008	*ΔtolA::tetRA, wza::lacZ::cat*	This study
ZD009	*ΔtolB::tetRA, wza::lacZ::cat*	This study
ZD010	*Δpal::tetRA, wza::lacZ::cat*	This study
ZD011	*ΔcpoB::tetRA, wza::lacZ::cat*	This study
ZD012	*ΔtolQ//tolQ*$^{+}$, *wza::lacZ::cat*	This study
ZD013	*ΔtolQ//tolQ*^{A177V}, *wza::lacZ::cat*	This study
ZD014	*ΔtolQ//tolQ*^{G181D}, *wza::lacZ::cat*	This study
Escherichia coli		
DH5-alpha	transformation and cloning intermediate	ATCC
**Plasmids**		
pBAD-empty	pBAD24 inducible expression vector	(3)
p*tolQ*	Encodes full length *tolQ*; Amp^R	This study
p*ybgC*	Encodes full length *ybgC*; Amp^R	This study
p*cpoB*	Encodes full length *cpoB*; Amp^R	This study
WT-pBAD	WT contains empty pBAD; Amp^R	This study
Δ*tolQ*-pBAD	Δ*tolQ* contains empty pBAD; Amp^R	This study
Δ*ybgC*-pBAD	Δ*ybgC* contains empty pBAD; Amp^R	This study
Δ*cpoB*-pBAD	Δ*cpoB* contains empty pBAD; Amp^R	This study
pBAV1K	Riboswitch controlled gene expression plasmid	(4)

pBAV1K-*tolQ*	Encodes full length *tolQ*; Kan^{R}	This study
pBAV1K-A177V *tolQ*	Encodes amino acid substitution on *tolQ* A177V	This study
pBAV1K-G181D *tolQ*	Encodes amino acid substitution on *tolQ* G181D	This study
pBAV1K-G181V *tolQ*	Encodes amino acid substitution on *tolQ* G181V	This study
pGRG37	pGRG37 transgene insertion plasmid; Amp^{R}	(5)
pGRG37-*tolQ*	Encodes full length *tolQ*; Amp^{R}	This study
pGRG37-A177V *tolQ*	Encodes amino acid substitution on *tolQ* A177V	This study
pGRG37-G181D *tolQ*	Encodes amino acid substitution on *tolQ* G181D	This study
pGRG37-G181V*tolQ*	Encodes amino acid substitution on *tolQ* G181V	This study

Table S2. Primers used in this study

Primer name	Primer sequence
ybgC::tetF	TACGGCGGCAAGCGGATGACGCCAACAAAGAGGCTGCAGGGTTAAGAACCCACTTTCACA
ybgC::tetR	GAAGGCTTGCCTTCAGGAACAAATCAAGGATATTCATGTCAGCTAAGCACTTGTCTCCTG
tolQ::tetF	CTTCCTAAGTCTATTGTCGCGGAGTTTAAGCAGTGACTGACTTAAGAACCCACTTTCACA
tolQ::tetR	CTTAAGTTCGCGACGACCTCGTCCACGCGTTCTGGCCATGGCCTAAGCACTTGTCTCCTG
tolR::tetF	CCAGGCGTTTACCGTAAGCGAAAGCAACAAGGGGTAAGCCTT AAGAACCCACTTTCACA
tolR::tetR	CCTGTTACTCGCCGTCTTTCAAGCCAACGGGACGCAGACTCTA AGCACTTGTCTCCTG
tolA::tetF	CTGCCGGCGGCGGCGGTGGTTCCGCTATCGACGCGGTGTTAAG AACCCACTTTCACA
tolA::tetR	GTAACAACTCAACTGCTCTAACTTCCATAAAGAAAAGTATCTA AGCACTTGTCTCCTG
tolB::tetF	TTAACATTCTGCTAAATTATCGTGGGTCGCAGGCCCAGGTTTAAGAACCCACTTTCACA
tolB::tetR	TTGCATTTCTTTAATTCCTTTAGTAATCAATTAATTATTACTAAGCACTTGTCTCCTG
pal::tetF	TCTGTGATAATAATTAATTGATTACTAAAGGAATTAAAGAATTAAGAACCCACTTTCACA
pal::tetR	CTCAACAGGTGATGTCTGAAGTTACTGCTCATGCAATTCTCCTAAGCACTTGTCTCCTG
cpoB::tetF	CTAAGAACCGTCGCGCTGTACTGGTTTACTAAGAGAATTGCTTAAGAACCCACTTTCACA
cpoB::tetR	ACGCGACCAGAAAAAAGGCGTTTTCTGGTCGCATGGTACGCACTAAGCACTTGTCTCCTG
pbavA*tolQ*	GCACCCTGCTAAGGAGGTAACAACAAGATGAATATCCTTGATTTGTTCCTGAA
pbavB*tolQ*	GCATCGATCGGGCCCTGAGGCCTGCAGTTACCCCTTGTTGCTTTCGCTTA
pbavC*tolQ*	TTCAGGAACAAATCAAGGATATTCATCTTGTTGTTACCTCCTTAGCAGGGTGC
pbavD*tolQ*	TAAGCGAAAGCAACAAGGGGTAACTGCAGGCCTCAGGGCCCGATCGATGC
pBAV FP *pac I*	GTTAATTAAAATTCGCGGCCGCTTCTAGA
pBAVRP *xhoI*	AACTCGAGGCCCTGAGGCCTGCAG
PM177A2VF	GCCGAAGCATTGATTATTACGGCGATTGGT
PM177A2VR	CATAACCGCAGGGATGGCGGCAAACAG
PM181Q2D	GATTGCCACGGCGATTGACCTGTTTGCCG

F	
PM181Q2DR	CGGTTGTAAGCCATAACCGCAGGGATGG
Primer A *tolQ*	GGAGGAATTCACCATGGTACCCGGGGATCCATGAATATCCTT GATTTGTTCCTGAA
Primer B *tolQ*	AGCCAAGCTTGCATGCCTGCAGGTCGACTCTAGA TTACCCCTTGTTGCTTTCGCTTA
Primer C *tolQ*	TTCAGGAACAAATCAAGGATATTCATGGATCCCCGGGTACCATGGTGAATTCCTCC
Primer D *tolQ*	TAAGCGAAAGCAACAAGGGGTAATCTAGAGTCGACCTGCAGGCATGCAAGCTTGGCT
Primer A *ybgC*	GGAGGAATTCACCATGGTACCCGGGGATCCATGAATAAGTATATGTTTCGATGGC
Primer B *ybgC*	AGCCAAGCTTGCATGCCTGCAGGTCGACTCTAGATCACTGCTTAAACTCCGCGAC
Primer C *ybgC*	TGCCATCGAAACATATACTTATTCATGATCCCCGGGTACCATGGTGAATTCCTCC
Primer D *ybgC*	GTCGCGGAGTTTAAGCAGTGATCTAGAGTCGACCTGCAGGCATGCAAGCTTGGCT
Primer A *cpoB*	GGAGGAATTCACCATGGTACCCGGGGATCCATGAGCAGTAACTTCAGACATCA
Primer B *cpoB*	AGCCAAGCTTGCATGCCTGCAGGTCGACTCTAGACATCGCGTTAAGACGCTTCT
Primer C *cpoB*	TGATGTCTGAAGTTACTGCTCATGGATCCCCGGGTACCATGGTGAATTCCTCC
Primer D *cpoB*	AGAAGCGTCTTAACGCGATGTCTAGAGTCGACCTGCAGGCATGCAAGCTTGGCT

Table S3. *tolQ*, *tolR*, and *tolA*-mutant STm do not measure significant differences in IM-GPL levels.

<u>Liquid-chromatography tandem mass spectrometry (LC-MS/MS) of IM-GPL molecules</u>

**m/z of GPL**	**WT IM**	***tolQ* IM**	***tolR* IM**	***tolA* IM**
PGl	ng/μl ± SD	ng/μl ± SD	ng/μl ± SD	ng/μl ± SD
719	80.18 ± 13.37	59.85 ± 5.33	75.08 ± 29.81	60.71 ± 8.64
733	4.94 ± 1.20	3.03 ± 0.22	5.12 ± 2.11	3.58 ± 0.52
747	9.12 ± 1.82	8.94 ± 0.37	13.17 ± 6.06	8.83 ± 1.22
773	11.85 ± 2.11	15.71 ± 0.07	22.71 ± 8.89	15.16 ± 1.02
PE	ng/μl ± SD	ng/μl ± SD	ng/μl ± SD	ng/μl ± SD
688	160.79 ± 14.92	140.21 ± 11.10	140.56 ± 11.95	134.44 ± 4.78
714	41.46 ± 4.56	45.34 ± 5.51	46.62 ± 4.88	41.99 ± 3.52
716	50.50 ± 4.19	55.88 ± 11.65	62.74 ± 9.80	52.41 ± 2.95
742	41.90 ± 5.20	59.17 ± 12.53	73.78 ± 18.32	54.93 ± 6.64

Overnight cultures of wild type, *tolQ*, *tolR*, and *tolA*-mutant STm were back diluted 1:100 in 1L of LB broth and incubated at 37°C and 225 rpm for ~ 3 h to the mid-exponential growth phase. The cells were collected by centrifugation and resuspended in a sucrose solution, which began the osmotic spheroplasting and lysis procedure. The membranes were isolated by discontinuous sucrose-density gradient ultracentrifugation (6). Three independent experiments were conducted and depicted are the average ng/μl values ± standard deviation (SD) for these biological replicates. m/z = mass to charge ratio, GPL= glycerophospholipid, PGl = phosphatidylglycerol, PE = phosphatidylethanolamine, SD = standard deviation from the mean.

Table S4. STm *ybgC* mutants accumulate PGls and PEs within the OM relative to the wild type.

<u>Liquid-chromatography tandem mass spectrometry (LC-MS/MS) of OM-GPL molecules</u>

**m/z of GPL**	**WT OM**	***ybgC* OM**
<u>PGl</u>	<u>ng/µl ± SD</u>	<u>ng/µl ± SD</u>
719	10.42 ± 2.39	19.49 ± 1.63*
733	1.30 ± 0.37	1.97 ± 0.31
747	2.35 ± 0.53	3.88 ± 0.34*
773	3.50 ± 0.74	8.21 ± 0.46*
		±
<u>PE</u>	<u>ng/µl ± SD</u>	<u>ng/µl ± SD</u>
688	104.08 ± 4.13	119.39 ± 7.94
714	9.14 ± 1.53	14.78 ± 1.45*
716	45.32 ± 4.98	51.29 ± 3.16
742	13.99 ± 3.26	24.69 ± 1.36*

Overnight cultures of wild type and *ybgC*-mutant STm were back diluted 1:100 in 1L of LB broth and incubated at 37°C and 225 rpm for ~ 3 h to the mid-exponential growth phase. Three independent experiments were conducted and depicted are the average ng/µl values ± standard deviation (SD) for these biological replicates. * Indicates a statistically significant difference for the mutant relative to the wild type $p < 0.05$. m/z = mass to charge ratio, OM = outer membrane GPL= glycerophospholipid, PGl = phosphatidylglycerol, PE = phosphatidylethanolamine, SD = standard deviation from the mean.

Table S5. STm *cpoB* mutants generally accumulate PGls and PEs within the OM relative to the wild type, but only minor significant differences are measureable.

Liquid-chromatography tandem mass spectrometry (LC-MS/MS) of OM-GPL molecules

**m/z of GPL**	**WT OM**	***cpoB* OM**
PGl	ng/µl ± SD	ng/µl ± SD
719	10.36 ± 2.00	17.50 ± 1.96
733	1.26 ± 0.29	1.88 ± 0.79
747	2.35 ± 0.53	4.17 ± 1.02
773	3.98 ± 1.71	7.05 ± 1.60
PE	ng/µl ± SD	ng/µl ± SD
688	68.14 ± 10.53	85.38 ± 4.13
714	10.04 ± 0.72	11.86 ± 0.80*
716	38.22 ± 5.92	48.74 ± 2.77
742	17.71 ± 3.19	27.06 ± 0.45*

Overnight cultures of wild type and *cpoB*-mutant STm were back diluted 1:100 in 1L of LB broth and incubated at 37°C and 225 rpm for ~ 3 h to the mid-exponential growth phase. Three independent experiments were conducted and depicted are the average ng/µl values ± standard deviation (SD) for these biological replicates. * Indicates a statistically significant difference for the mutant relative to the wild type $p < 0.05$. m/z = mass to charge ratio, GPL= glycerophospholipid, PGl = phosphatidylglycerol, PE = phosphatidylethanolamine, SD = standard deviation from the mean.

Table S6. STm *tol-pal* mutants are shorter than the wild type.

Genotype	Length (µm)	Width (µm)
WT	1.31±0.24	0.32±0.08
ΔybgC	1.01±0.29	0.23±0.01
ΔtolQ	0.85±0.23	0.46±0.11
ΔtolR	0.87±0.30	0.41±0.08
ΔtolA	0.88±0.27	0.37±0.10
ΔtolB	0.93±0.27	0.39±0.08
Δpal	0.94±0.34	0.36±0.08
ΔcpoB	0.98±0.13	0.35±0.11

Exponential-phase bacteria were fluorescently labeled with the lipophilic membrane dye, FM4-64, before being spotted onto agarose pads and visualized by phase contrast and epifluorescence microscopy at 100X magnification. To measure the cells, we used the fluorescently outlined bacteria from the FM4-64 images and the ImageJ software. The control scale was set using the analyze > set scale option. The cell length and width were measured using the line tool and the results were recorded. Three different fields and a total of fifty cells were measured for each strain. The average ± SD for each genotype is shown.

SUPPORTING FIGURES

Figure S1.

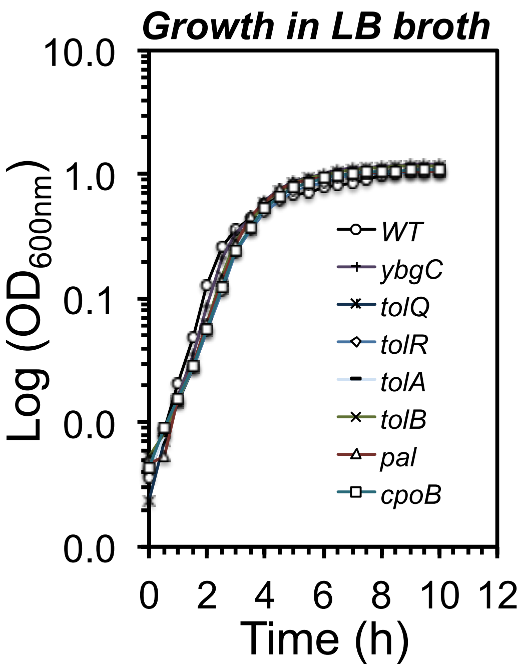

Fig. S1. *tol-pal* mutants divide at rates comparable to the wild-type STm in Luria-Bertani (LB) broth media. Bacteria were cultured overnight (OVN) in LB broth, normalized to an OD_{600nm} of 0.01, and distributed between triplicate wells of a ninety-six well plate. The plate was incubated with aeration at 37°C in a growth curve analyzer. Optical density was recorded as a function of time over 0.5 h intervals until the bacteria reached the stationary growth phase. Depicted is the average ± SD for the three individual wells for each time point.

Figure S2.

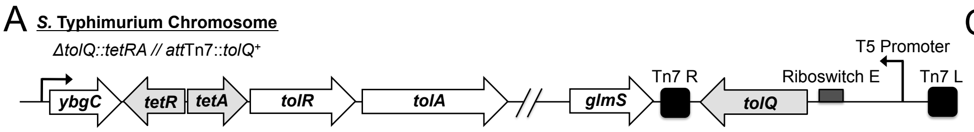

B

Amino acid alignment for the three transmembrane (TM) domains of the three ion-channel proteins

TM1	STm TolQ	L	L	**V**	**K**	L	I	**M**	L	I	**L**	**I**	G	F	**S**	I	A	S	**W**	**A**	**I**	I
TM1	STm ExbB	I	V	**V**	**K**	C	V	**M**	I	G	**L**	**I**	L	A	**S**	V	V	T	**W**	**A**	**I**	F
TM1	*E. coli* TolQ	L	L	**V**	**K**	L	I	**M**	L	I	**L**	**I**	G	F	**S**	I	A	S	**W**	**A**	**I**	I

TM2	STm TolQ	Y	I	**G**	**L**	**F**	**G**	**T**	**V**	**W**	**G**	**I**	**M**	H	A	**F**	**I**
TM2	STm ExbB	F	V	**G**	**L**	**F**	**G**	**T**	**V**	**W**	**G**	**I**	**M**	N	S	**F**	**I**
TM2	*E. coli* TolQ	Y	I	**G**	**L**	**F**	**G**	**T**	**V**	**W**	**G**	**I**	**M**	H	A	**F**	**I**

TM3	STm TolQ	I	A	E	**A**	**L**	I	**A**	**T**	**A**	**I**	**G**	**L**	P	**A**	**A**	**I**	**P**	**A**	**V**	M	A	**Y**
TM3	STm ExbB	-	-	-	**A**	**L**	L	**A**	**T**	**A**	**I**	**G**	**L**	V	**A**	**A**	**I**	**P**	**A**	**V**	V	I	**Y**
TM3	*E. coli* TolQ	I	A	E	**A**	**L**	I	**A**	**T**	**A**	**I**	**G**	**L**	P	**A**	**A**	**I**	**P**	**A**	**V**	M	A	**Y**

C

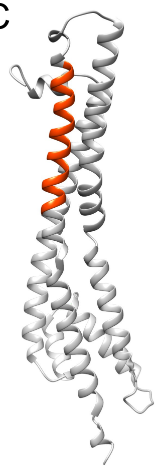

Fig. S2. Schematic depicting the *tolQ*-mutant complementation strategy, which we used to test the functional role of conserved TolQ channel residues within the third transmembrane (TM) helix of the protein. A. Schematic representation of the complementation genotype that was engineered by cloning *tolQ* alleles at the *att*Tn7 integration site, which is 3' of *glmS* on the STm genome (5). The *tolQ* alleles were expressed from a constitutive T5 promoter. Translation was repressed by riboswitch E (4, 7). Riboswitch repression can be relieved to regulate TolQ translation by adding the small-molecule inducer theophylline (Table S1 and S2). **B.** Shown is an alignment of the consensus sequence for the three putative transmembrane (TM) domains of STm 14028s TolQ and ExbB, and *E. coli* K-12 MG1655 TolQ. Conserved residues are shown in bold. Conserved TM3 residues that directly facilitate *E. coli* TolQ ion-transport activity are highlighted in yellow. These residues were tested for functional involvement in the present study by constructing substitution mutant alleles of TolQ at these amino acids. **C.** Shown is a three-dimensional model of the structure for STm TolQ. This model was predicted using the I-TASSER server and the known structure of the ExbB/ExbD complex (PDB: 5SVO) as a template. TM3 is colored orange.

REFERENCES

1. Dalebroux ZD, Matamouros S, Whittington D, Bishop RE, Miller SI. 2014. PhoPQ regulates acidic glycerophospholipid content of the Salmonella Typhimurium outer membrane. Proc Natl Acad Sci U S A 111:1963-8.
2. Farris C, Sanowar S, Bader MW, Pfuetzner R, Miller SI. 2010. Antimicrobial peptides activate the Rcs regulon through the outer membrane lipoprotein RcsF. J Bacteriol 192:4894-903.
3. Guzman LM, Belin D, Carson MJ, Beckwith J. 1995. Tight regulation, modulation, and high-level expression by vectors containing the arabinose PBAD promoter. J Bacteriol 177:4121-30.
4. Topp S, Reynoso CMK, Seeliger JC, Goldlust IS, Desai SK, Murat D, Shen A, Puri AW, Komeili A, Bertozzi CR, Scott JR, Gallivan JP. 2011. Synthetic Riboswitches That Induce Gene Expression in Diverse Bacterial Species (vol 76, pg 7881, 2010). Applied and Environmental Microbiology 77:2199-2199.
5. McKenzie GJ, Craig NL. 2006. Fast, easy and efficient: site-specific insertion of transgenes into enterobacterial chromosomes using Tn7 without need for selection of the insertion event. BMC Microbiol 6:39.
6. Castanie-Cornet MP, Cam K, Jacq A. 2006. RcsF is an outer membrane lipoprotein involved in the RcsCDB phosphorelay signaling pathway in Escherichia coli. J Bacteriol 188:4264-70.

7. Topp S, Reynoso CM, Seeliger JC, Goldlust IS, Desai SK, Murat D, Shen A, Puri AW, Komeili A, Bertozzi CR, Scott JR, Gallivan JP. 2010. Synthetic riboswitches that induce gene expression in diverse bacterial species. Appl Environ Microbiol 76:7881-4.
